# Role and regulatory mechanism of microRNA mediated neuroinflammation in neuronal system diseases

**DOI:** 10.3389/fimmu.2023.1238930

**Published:** 2023-08-11

**Authors:** Jingdan Zhang, Ao Li, Runze Gu, Yueyang Tong, Jinbo Cheng

**Affiliations:** Center on Translational Neuroscience, College of Life and Environmental Science, Minzu University of China, Beijing, China

**Keywords:** miRNA, neuroinflammation, Alzheimer’s disease (AD), stroke, TBI, therapy

## Abstract

MicroRNAs (miRNAs) are small non-coding RNAs with the unique ability to degrade or block specific RNAs and regulate many cellular processes. Neuroinflammation plays the pivotal role in the occurrence and development of multiple central nervous system (CNS) diseases. The ability of miRNAs to enhance or restrict neuroinflammatory signaling pathways in CNS diseases is an emerging and important research area, including neurodegenerative diseases, stroke, and traumatic brain injury (TBI). In this review, we summarize the roles and regulatory mechanisms of recently identified miRNAs involved in neuroinflammation-mediated CNS diseases, aiming to explore and provide a better understanding and direction for the treatment of CNS diseases.

## 1 Introduction

In 1993, a new class of small RNA molecules called miRNAs was first discovered in *C. elegans* (1). MiRNAs are non-protein-coding RNAs that can degrade or sequester specific RNA, prevent protein translation, and regulate myriad cellular processes (2). As reported, more than 5,000 miRNAs can target up to 80% of protein-coding genes, each of which regulates the translation of hundreds of different messenger RNAs (mRNAs) (3–6). Therefore, it is important to understand how specific miRNAs regulate cellular processes. In this review, we summarize how miRNAs affect inflammatory signaling pathways and review their functions in the treatment and prognosis of neurological diseases.

Nervous system inflammation is a positive response caused by autoimmune defense against injury; however, it usually aggravates pathological injury (7–10). In models of CNS trauma, neuroinflammation can aggravate the injury and cause secondary injuries (11–14). Therefore, it should take advantage of the beneficial aspects of inflammation while limiting its overreaction to aggravate the pathology and subtly improve neurological function. In the immune inflammatory response of CNS diseases, especially ischemic stroke (IS), intracerebral hemorrhage (ICH), neurodegenerative diseases, and TBI, miRNAs have been reported to promote or inhibit the nuclear factor kappa-B (NF-κB) signaling, mitogen-activated protein kinases (MAPK) signaling, and NOD-like receptor thermal protein domain associated protein 3 (NLRP3) inflammasome, and these signaling pathways are highly correlated with proinflammatory response. Therefore, exploring the roles and regulatory mechanisms of miRNAs in CNS diseases would be beneficial for drug discovery and targeting therapies.

## 2 miRNA biogenesis and function

MiRNAs are one type of small RNAs encoded by endogenous genes, which usually contain 20-24 nucleotides and regulate the post-transcriptional gene expression. The biogenesis of miRNA has been well described in several reviews (15, 16).

**Figure 1:**
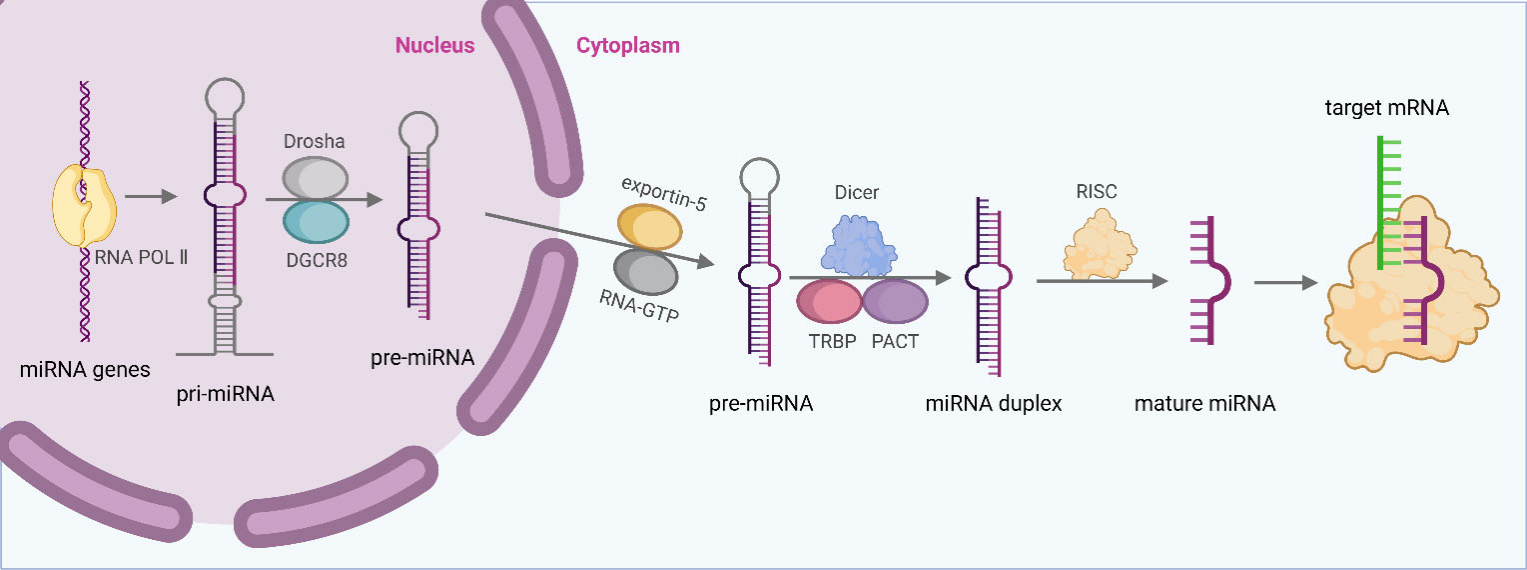
MiRNA biogenesis and function. Generally, miRNA first transcribe longer pri-miRNA, and pri-miRNA are then cleaved to pre-miRNA by Drosha and Pasha/DGCR8 in the nucleus. Subsequently, the pre-miRNAs are transported to cytoplasm with the help of the Exprotin-5 complex, and cleaved by Dicer into mature miRNAs. Finally, with the help of miRISC, mature miRNAs binds to 3′-UTRs of target mRNAs and regulate gene expression.

In animal cells, miRNA first transcribe longer primary miRNA (pri-miRNA) in the nucleus. The pri-miRNA are then cleaved to be precursors (pre-miRNA) by Drosha and Pasha/DGCR8 in the nucleus (15, 17). Subsequently, the pre-miRNAs are transported out of the nucleus with the help of the Exprotin-5 complex, and cleaved by Dicer into mature miRNAs of 21-25 nucleotides in the cytoplasm. Finally, with the help of miRNA-induced silencing complex (miRISC), mature miRNAs binds to 3′-untranslated regions (3′-UTRs) of target mRNAs and regulate gene expression (17, 18) (**Figure 1**).

The presence of miRNAs was first discovered in *C. elegans*. They can bind to the 3′-UTR of the target mRNA through partial complementarity, inducing protein translation inhibition. With the deepening of miRNA research, scientists are conscious of these small molecules holding a variety of functions. Additionally, the expression levels of miRNAs vary significantly at different time, suggesting that miRNAs are crucial in regulating gene expression (19).

## 3 Inflammation contributes to neuropathology

Neuroinflammation refers to the inflammatory response in the CNS caused by pathological injuries, such as infection, trauma, ischemia, and toxin accumulation, which can also lead to disease exacerbation. As an immune-privileged tissue, CNS parenchyma is generally not exposed to peripheral immune cells or robust inflammatory responses in healthy condition. In the CNS of healthy adults, microglia and astrocytes remain quiescent and undergo immune surveillance. Upon infection or injury, these cells transiently activate and increase inflammation. The activated astrocytes and microglia trigger inflammatory response by producing multiple chemokines and cytokines, such as chemokine ligand (CCL1, CCL5), interleukin (IL-1β, IL-6, IL-18), tumor necrosis factor (TNF), and small molecule messengers (prostaglandins, NO, reactive oxygen species). These factors contribute to the inflammatory response and subsequent restoration of CNS homeostasis (20–22). Therefore, targeting inflammation-related signaling pathways is a beneficial direction for the treatment of CNS diseases.

Our previous work has shown that microglia Calhm2 played a vital role in microglia activation, not only in chronic inflammatory disease, but also in acute inflammatory responses (23). Knockout of Calhm2 reduced the activation of NLRP3 inflammasome. Mechanistically, Calhm2 not only regulated NF-κB signaling pathway, but also regulated the interaction between P2X7 and NLRP3. As a cationic channel, P2X7 could regulate NLRP3 inflammasome activation, and highly expressed in microglia (24–26). Recently, we found that Calhm2 played a critical role in Parkinson’s disease (PD) by regulating EFhd2 expression in microglia (27). Moreover, in our another study, we found that 1,2,4-Trimethoxybenzene (1,2,4-TTB) could inhibit NLRP3 inflammasome activation to reduce clinical symptoms and inflammation in experimental autoimmune encephalomyelitis (EAE) (28), suggesting that 1,2,4-TTB might be used in the treatment of inflammatory diseases driven by NLRP3 inflammasome. Moreover, NLRP3 inflammasome activation was also associated with the development of posttraumatic stress disorder (PTSD). Inhibition of the NLRP3 inflammasome remarkably attenuated PTSD-like behavior in mice (29). Besides of NLRP3 inflammasome, NF-κB was another most studied inflammatory factor, which was a prominent transcription factor that aggravated neuroinflammation and could promote the transcription of a range of inflammatory mediators. Our studies showed that conditional deletion of microglial Dlg1 significantly inhibited the NF-κB signaling activation in microglia, and alleviated lipopolysaccharides (LPS)-induced and chronic restraint stress-induced depression-like behaviors in mice (30, 31). Moreover, treatment of Bergapten inhibited microglial activation and alleviated LPS-induced depression-like behavior in mice (32). Collectively, microglial activation-induced neuroinflammation was critical in the development of these diseases, and targeting neuroinflammation might be a promising strategy in the clinical treatment.

**Figure 2:**
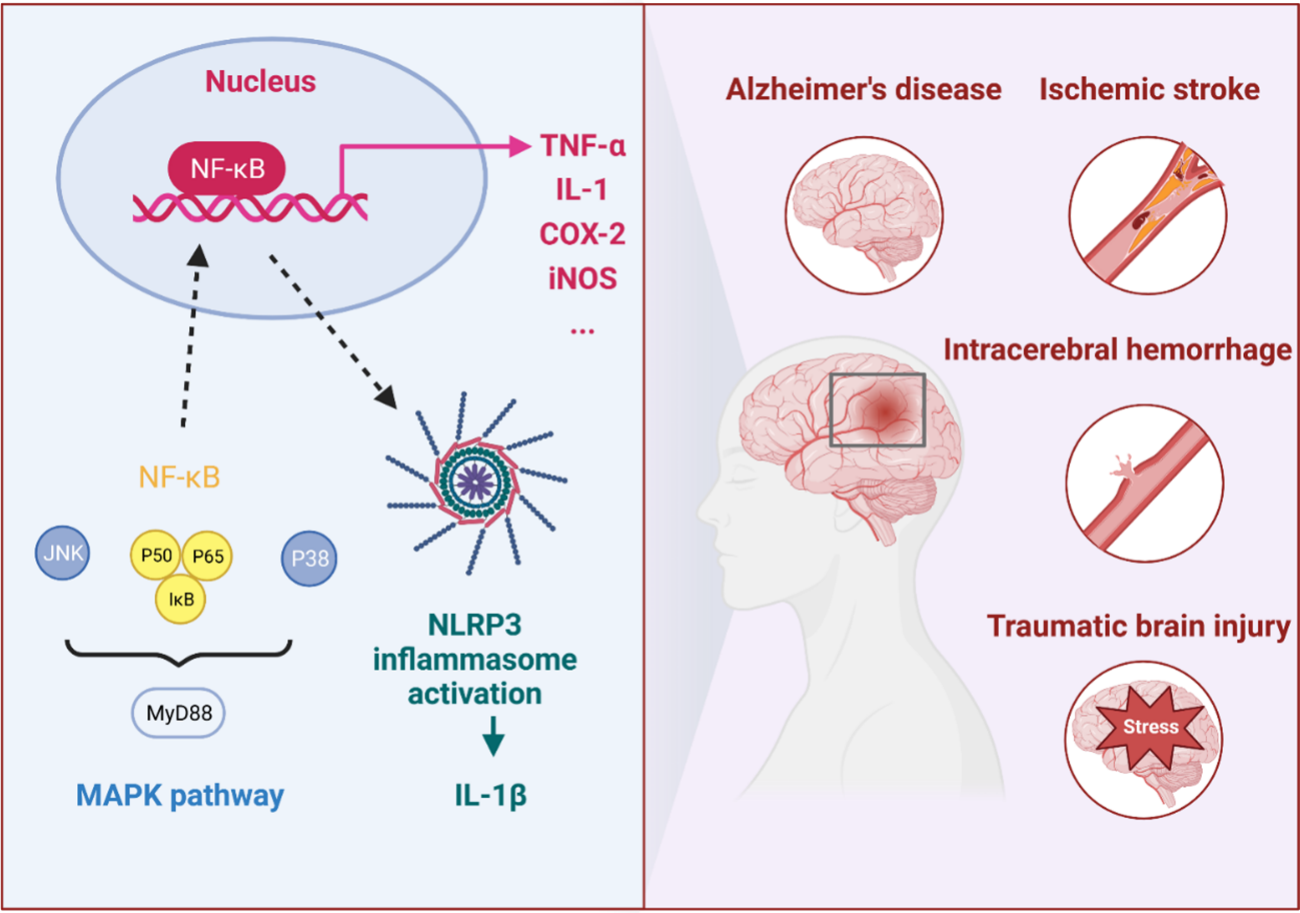
The immune inflammatory response in CNS diseases. In the immune inflammatory response of CNS diseases, especially in IS, ICH, AD and TBI, the NF-κB and MAPK signaling pathways and NLRP3 inflammasome were highly correlated with proinflammatory response.

It was reported that miRNAs could reduce the inflammatory response by inhibiting TLR4 and the downstream MyD88/TRIF/NF-κB inflammatory pathway (33). Moreover, miRNAs targeting on the upstream and downstream of MAPK signaling pathway could also reduce the inflammatory response. Increased MAPK expression was important for the activation of inflammatory processes, including three subfamilies: p38 mitogen-activated protein kinase (p38), c-Jun-terminal kinase (JNK), and extracellular signal-regulated kinase 1/2 (ERK1/2) (34). Therefore, miRNA was closely associated with multiple inflammatory signaling pathways. Here, we review the functions and inflammatory targets of miRNA in different CNS diseases, aiming to understand the underlying mechanism and emphasize the therapeutic application of miRNAs (**Figure 2** and **Table 1**).

**Table 1:** The miRNAs associated with neuroinflammation-related neurological diseases.

MicroRNA	Expression pattern	Related diseases	Target/Signaling pathway	Reference
miR-29a	down	AD	TNF-α/NF-кB	(35, 36)
miR-34a	up	AD	TREM2/NF-кB	(37–39)
miR-126-3p	up	AD	VCAM-1/NF-кB	(40, 41)
miR-146a	up	AD	IRAK-1/NF-кB	(42)
miR-155	up	AD, IS, ICH	c-Jun/NF-кB, TLR4/MyD88/NF-кB, BMAL1/Nrf2	(43–47)
miR-132	up	AD	MAPK1/MAPK	(48)
miR-223-3p	down	AD	NLRP3	(49–55)
miR-101	down	AD	COX2	(56, 57)
miR-181	up, down	AD, IS	c-Fos, CXCL1	(58–61)
miR-22	down	IS	p38 MAPK/NF-κB	(62)
miR-195	down	IS, ICH	CD40/NF-κB, CX3CR1, IKKα/NF-κB	(63–65)
miR-203	–	IS	MyD88/NF-κB	(66)
miR-1202	–	IS	TLR4/Rab1A/NF-κB	(67)
miR-20b	up	IS	NLRP3	(68)
miR-140-5p	–	ICH	MyD88/TRIF/NF-κB	(33)
miR-181c	down	ICH	MLL1/NF-κB	(69)
miR-152	down	ICH	TXNIP/NLRP3	(70)
miR-194-5p	down	ICH	TRAF6/NLRP3	(71)
miR-223	–	ICH	NLRP3	(72)
miR-183-5p	down	ICH	HO-1/Nrf2	(73)
miR-21-5p	up	ICH, TBI	p-ERK/HO-1, NF-κB	(74, 75)
miR-132	down	ICH	AChE	(76)
miR-590-5p	down	ICH	Peli1	(77)
miR-23a-3p	down	TBI	AKT/mTOR	(78)
miR-200b	down	TBI	cJun/MAPK	(79)

## 4 miRNA related inflammatory responses in CNS diseases

### 4.1 miRNA related inflammatory responses in AD

As an irreversible progressive neurodegenerative disease, AD is the most common form of dementia in the elderly, accounting for 80% of all diagnoses (80). Progressive deteriorated memory and other cognitive abilities are clinical hallmarks, eventually leading to an inability to live independently (81). The most known theory about the pathogenesis of AD is the Aβ toxicity hypothesis. Aβ aggregation (Aβ42) is thought to be a major mediator and crucial driver in the pathogenesis of AD (82, 83). Moreover, Aβ is considered a neurotoxic protein that causes an inflammatory response which accelerates cell death (84, 85). Therefore, the onset and progression of AD is closely associated with the activation of the brain’s inflammatory response. Robust immune responses following Aβ stimulation may lead to undifferentiated damage of healthy neural tissues, resulting in neuronal cell damage and cell death (86). Additionally, the blood-brain barrier (BBB) fails to prevent the entry of lymphocytes and inflammatory cytokines into the brain is another main cause of inflammatory response. MiRNAs exert negative regulatory effects by interacting with 3’‐UTR of target mRNA, degrading mRNA or inhibiting protein translation. Moreover, miRNAs can influence the inflammatory responses in AD, which are closely associated with the onset and progression of AD.

#### 4.1.1 miRNAs in NF-κB signaling pathway in AD

MiR-29a, miR-29b, and miR-29c were belong to the miR-29 family, and the levels of miR-29a and miR-29b-1 were decreased in AD patients (87). Srivastava et al. (2016) predicted miR-29a could target key members of the TNF-mediated pathway, including TRAF3, TRAF4, TRAF5, TNFRSF1A, LTBR, TNFAIP1, and TNFAIP3 (35). Moreover, Sedighi et al. (2019) reported that TNF-α receptor expression in AD was negatively correlated with miR-29a levels. Therefore, miR-29a may be associated with AD by regulating the neuroinflammation levels (36).

MiR-34a, a miRNA regulated by NF-кB, was upregulated in the hippocampal CA1 region of AD patients. Bhattacharjee et al. (2016) found that miR-34a modulated TREM2 (a microglial receptor that triggers expression in myeloid cells), a crucial molecule of Aβ42 peptide clearance in AD. Downregulation of TREM2 levels was observed in the CA1 region in AD patients (37, 38). Rare variants R47H of TREM2 have been linked to an increased risk of AD, indicating that targeting miR-34a might be a therapeutic strategy for AD treatment (39).

MiR-126 regulated inflammation by targeting NF-κB pathway components and vascular cell adhesion molecule 1 (VCAM-1). The VCAM-1 mRNA was not expressed in quiescent endothelium cells, but pro-inflammatory cytokines could activate NF-κB and IRF-1, thereby inducing the transcription of VCAM-1. Overexpression of miR-126 could decrease VCAM-1 levels, suggesting miR-126 regulated VCAM-1 levels in quiescent cells (40). Moreover, elevated levels of miR-126 increased Aβ42-induced toxicity and interfered with the neuroprotective effects of IGF-1 by inhibiting PI3K and ERK pathways (41), suggesting miR-126 may be a potential molecular target for AD treatment.

MiR-146a was another NF-κB-dependent miRNA, that targeted transmembrane β-amyloid precursor protein (APP) associated TSPAN12, and inflammatory mediators interleukin receptor-associated kinase IRAK-1 (42). Therefore, miRNA-146a could be used as a potential diagnostic biomarker for AD and other age-related neurological disorders.

MiR-155 was also a known target for NF-кB pathway. Guedes et al. (2014) found that miR-155 was increased in the brains of AD animals. Moreover, the levels of miR-155 depended on the transcription factor c-Jun, which preceded extracellular Aβ accumulation and concomitant hyperactivation of microglia and astrocytes (43). Interestingly, Song and Lee (2015). found that miR-155 could regulate T lymphocyte function, suggesting that miR-155 was an immune-related miRNA associated with AD (44). Therefore, miR-155 may not only be a valuable candidate biomarker of AD, but also a therapeutic target for AD.

#### 4.1.2 miRNAs in MAPK signaling pathway in AD

It has been reported that miR-132 improved cognitive function in AD rats by inhibiting the MAPK1 signaling pathway. Bioinformatics analysis revealed a target inhibitory relationship between miR-132 and MAPK1. It was found that upregulation of miR-132 reversed the negative effects of MAPK1 silencing in AD rats. Mechanistically, miR-132 inhibited the expression of iNOS and oxidative stress in the hippocampus by inhibiting the expression of MAPK1, and improved the cognitive function of AD rats, which was helpful for understanding the pathogenesis of AD and developing new clinical treatments (48).

#### 4.1.3 miRNAs in NLRP3 signaling pathway in AD

Multiple studies have showed that some miRNAs, including miR-223, were dysregulated in AD and played important roles in AD pathogenesis (49, 50). MiR-7-5p, miR-22-3p, and miR-30-5p could bind to the NLRP3-mRNA, hindered protein translation, and prevented the formation of inflammasome protein complexes. In the brains of AD patients, other studies have also found downregulation of miR-7 and miR-30e as well as low levels of circulating miRNA-22 (51, 52). La Rosa et al. (2021) analyzed the expression of miRNAs in AD patients, and found that miR-223-3p and miR-7-5p levels were increased in AD patients, but failed to downregulate NLRP3 and proinflammatory cytokines (53). In particular, miR-223-3p bound to the highly conserved 3’UTR of NLRP3 and acted as a NLRP3 miRNA inhibitor (54, 55).

#### 4.1.4 miRNAs in other inflammatory molecular pathways in AD

MiR-101 was downregulated in temporal and parietal regions of human AD cortex (56). Downregulation of miR-101 leaded to upregulation of COX2 in AD and induced inflammatory responses in the brain, thus significantly promoting AD pathology (57).

The miR-181 family was also involved in neuroinflammation and is associated with AD (58). Rodriguez-Ortiz et al. (2014) found that miR-181a was significantly upregulated in the hippocampus of 12-month-old AD mice. Enhancement of the expression of miR-181 in SH-SY5Y cells significantly reduced SIRT1 and c-Fos levels (59). Moreover, miR-181 family have been linked to the stress responses, in which miR181d was discovered to target multiple stress and metabolic related signaling pathway. Collectively, these results suggest a possible between the miR-181 family in stress response and neuroinflammation in AD (60).

Together, miRNAs could participate in the regulation of MAPK pathway, NF-κB signaling, NLRP3 inflammasome, and other inflammatory molecular pathways to regulate neuroinflammation. Among them, NF-κB signaling related miRNAs include miR-29 family, miR-34a, miR-126-3p, miR-146a and miR-155. These miRNAs may not only be valuable candidate biomarkers but also therapeutic targets for AD.

### 4.2 miRNAs related neuroinflammation in ischemic stroke

Ischemic stroke (IS) is a neurovascular event with high morbidity, mortality, and disability and is a great threat to the health of society and individuals. Currently, stroke is mainly cause of mortality in China and the second leading of death in the world (88–90). Neuroinflammation is an important hallmark of IS. In IS, interactions between inflammatory and non-inflammatory cells, brain cells such as neurons, are complex and extensive, with both deleterious and beneficial outcomes (91). With extensive research on neuroinflammation and IS, miRNAs have been recognized as promising novel regulators of neuroinflammation associated with IS (92). Notably, the relative stability, specificity and reproducibility make miRNAs possible to become good biomarkers in clinical treatment of diseases. Here, we summarize the miRNAs associated with neuroinflammation in IS.

#### 4.2.1 miRNAs in NF-κB signaling pathway in IS

MiR-22 alleviated inflammation in IS by inhibiting the p38 MAPK/NF-κB pathway (62). Dong et al. (2019) found that downregulation of miR-22 upregulated inflammatory factors and overexpression of miR-22 decreased inflammatory factors expression *in vitro*, suggesting that targeting miR-22 might be beneficial for the prevention and treatment of IS.

MiR-155 participated in cell damage by regulating the expression of TLR4 and MyD88, which might be important for the diagnosis and treatment of IS. Moreover, the miR-155 expression level was found to be higher in the serum of IS patients, but it decreased after treatment (45). Chen et al. (2021) found that the knockout of miR-155 improved the neurological function of mice and inhibited TLR4 and MyD88 protein levels. Additionally, miR-155 inhibition enhanced the proliferation of SH-SY5Y cells, reduced apoptosis levels, and increased the expression of TLR4 and MyD88. Interestingly, treatment with TLR4/MyD88 pathway inhibitors completely reversed the effects of miR-155 (46), indicating that miR-155 could regulate disease by targeting the TLR4/MyD88 pathway in IS.

Yang et al. (2021) found that the decreased expression of miR-195 and increased NF-κB expression were occurred in oxygen–glucose deprivation (OGD)-treated HUVECs. Importantly, miR-195 overexpression inhibited apoptosis and promoted cell proliferation by regulating IKKα-mediated NF-κB signaling pathway, suggesting that miR-195 inhibited the IKKα-mediated NF-κB pathway to have a protective role, offering a novel possible strategy for the clinical treatment of IS (63). In addition, CD40, a protein expressed in nerve and blood vessel cells in the brain, could stimulate the NF-κB pathway, but its activity could be directly inhibited by miR-195. CD40 levels increased in the cerebral apoplexy hemisphere, and its level was associated with the post-ischemic inflammation. CD40 deficiency reduced cell adhesion, reduced NF-κB signaling pathway, and increased iNOS level. Intravenous injection of nanoparticle-carried miR-195 into rats at the acute stage of IS reduced the volume of the injured brain by up to 45% and improved functional recovery (64), suggesting that treatment with miR-195 was beneficial for treating acute IS, which might act as a new target of IS.

MiR-203 protected microglia-mediated brain injury by inhibiting NF-κB signaling pathway during ischemia. Z. Yang et al. (2015b) found that miR-203 directly bound to the 3’UTR of MyD88 to inhibit its expression, which suppressed neurological inflammatory response and improved neuronal functions (66). This finding suggests that miRNA-203 is a novel target that can attenuate inflammatory response in IS and reduce neuronal damage.

Overexpression of miR-1202 inhibited the activation of the TLR4/NF-κB inflammatory signaling pathway, thereby exerting a neuroprotective effect. Song et al. (2020) found that miR-1202 expression was downregulated and Rab1a expression was upregulated in OGD/R-induced human microglial cell line. Rab1a (NF-κB upstream signaling protein) could up-regulate the expression level of TLR4 in OGD/R-treated cells, and promoted the activation of NF-κB signaling pathway (67). Interestingly, miR-1202 directly targeted Rab1a to inhibit NF-κB signaling pathway, and reduced inflammatory response and apoptosis, exhibiting a protective effect.

#### 4.2.2 miRNAs in NLRP3 signaling pathway in IS

Reportedly, miR-20b was involved in cerebral ischemia-induced inflammation by targeting NLRP3. Downregulation of miRNA-20b inhibited the NLRP3 signaling pathway and the downstream IL-1β and IL-18 levels, and reduced ATP and ROS levels, thereby mitigating inflammatory damage after IS (68).

#### 4.2.3 miRNA in other inflammatory molecular pathways in IS

MiR-181 was highly expressed in healthy human brains and was downregulated in acute ischemic stroke (AIS) patients. Ma et al. (2016) found that downregulated miR-181c-3p levels observed in OGD-treated cortical neurons and OGD-treated exosomes. CXCL1 and inflammatory cytokine expression in astrocytes were decreased by OGD-treated cortical neuronal exosomes. Moreover, the miR-181c-3p mimics against CXCL1 inhibited astrocytic inflammation levels by downregulating CXCL1 (61).

In addition, the expression of miR-195 was significantly downregulated in the plasma samples of AIS patients, with a significant negative correlation with Stroke Scale score. Consistently, miR-195 levels were also decreased in the plasma in MCAO mice, and intracerebral injection of miR-195 lentivirus inhibited inflammatory signal transduction. Mechanistically, the authors found that miR-195 could directly target both CX3CL1 and CX3CR1, and inhibited CX3CR1-mediated neuroinflammation (65).

In conclusion, miRNAs can directly or indirectly regulate several inflammatory pathways, such as NF-κB signaling pathway and NLRP3. Targeting the regulation of miRNA facilitates the diagnosis and treatment of IS.

### 4.3 miRNAs related neuroinflammation in ICH

ICH is associated with massive hemorrhage, resulting in decreased regional cerebral blood flow, inflammation, and immune responses (93), which has two types-primary and secondary ICH. Its pathogenic mechanism is complex and diverse; however, most ICH cases cause devastating damage to patients (94). Primary injury is mainly caused by the rapid formation of hematomas and hematoma mass effect caused by physical damage. Secondary damage evolves into an overlapping continuum with primary damage, caused by extravasation of blood components and associated neurotoxicity. Neuroinflammation, oxidative stress, apoptosis, and excitotoxicity are involved in the secondary brain damage (95).

Reportedly, therapeutically targeting neuroinflammation can modulate the potential for brain injury and repair following ICH. Peripheral immune cells are rapidly activated after vascular extravasation, releasing a series of toxic mediators and triggering an inflammatory cascade reaction. Both immediate local damage and long-term impairment of brain function can be induced by neuroinflammatory reactions (96). Additionally, miRNAs could regulate neuroinflammation after ICH by affecting various inflammatory components (97). The discovery of the function and regulatory mechanism of miRNAs in ICH is beneficial for the exploration of new strategy in the clinical therapies.

#### 4.3.1 miRNAs in NF-κB signaling pathway in ICH

It was reported that overexpression of miR-140-5p decreased TLR4 levels, and inhibited MyD88/TRIF/NF-κB inflammatory signaling pathway, thereby reduced brain injury and neuroinflammation after ICH (33), suggesting that targeting miR-140-5p is beneficial to therapeutic treatment of ICH.

Moreover, thrombin-induced downregulation of miR-181c promoted MLL1 levels, and increased the activity of NF-κB signaling (69). These results suggest that proinflammatory NF-κB activity stimulated by thrombin is involved in the pathology of ICH, which could be inhibited by overexpression of miR-181c. Therefore, miR-181c mimic therapy holds promise for regulating thrombin-driven microglial activation after ICH.

MiR-195 inhibited NF-κB signaling, including IKKα and p-IκB, which could affect the reduction of ubiquitin-dependent IκB degradation, leading to the inhibition of nuclear translocation of p65/p50 and RelB/p52, and downregulation downstream pro-inflammatory cytokine levels in the rat brain, suggesting that miR-195 has anti-inflammatory effects. Moreover, experimental results showed that miR-195 carried by nanoparticles and injected intravenously into rats at the acute stage of hemorrhagic stroke could reduce about 30% of the damaged brain volume (64). In conclusion, miR-195 could be used to treat ICH, since it directly blocks the NF-B pathway to exhibit its anti-inflammatory effects.

#### 4.3.2 miRNAs in NLRP3 signaling pathway in ICH

MiR-152 regulated the thioredoxin-interacting protein (TXNIP)-mediated NLRP3 activation, and inhibited neuroinflammation and neuronal death after ICH. Previous studies have shown that downregulated miR-152 was observed in ICH patients. Hu et al. (2020) found that miR-152 was downregulated in both collagenase-induced rat ICH model and hemin exposure model. Overexpression of miR-152 significantly alleviated hematoma, brain edema, and neurological deficits in rats with ICH. Mechanistically, overexpression of miR-152 blocked the interaction between TXNIP and NLRP3, and inhibited the activation of NLRP3 inflammasome (70), indicating that miR-152 played an active role in NLRP3 activation.

Wan et al. (2021) found that injecting miR-194-5p agomir into the brain tissue significantly inhibited NLRP3-mediated inflammation and alleviated the neuropathological damage in ICH rats. TRAF6 was further discovered to be one target of miR-194-5p. Overexpression of miR-194-5p reduced the interaction between NLRP3 and TRAF6, thereby reducing NLRP3 inflammasome-mediated neuroinflammation (71).

Moreover, miR-223 could directly bind to the 3’UTR of mRNA of NLRP3, and inhibit NLRP3 expression, thereby reducing neuronal inflammation and improving neuronal function. The pathology of ICH was characterized with inflammation, nerve damage, and increase of brain water content in mice, and miR-223 could reduce these changes by down-regulation of NLRP3 inflammasome (72).

#### 4.3.3 miRNAs in Nrf2 signaling pathway in ICH

Inhibition of miRNA-155 promoted the BMAL1 levels, which further activated the nuclear factor erythroid 2-related f actor 2 (Nrf2) to alleviate ICH-induced secondary brain injury (SBI). Gong et al. (2021) established a rat model of ICH using autologous blood injections and found that BMAL1 protein levels were decreased in the ICH group. Moreover, ICH-induced oxidative stress, inflammation, brain edema, BBB damage, neuronal cell death, and neurological dysfunction were alleviated by overexpressing of BMAL1 (47). Therefore, the upregulation of BMAL1 could activate the Nrf2 signaling pathway to attenuate SBI after ICH, and the miR-155/BMAL1 might be a promising therapeutic target.

Overexpression of miR-183-5p reduced HO-1 expression and thus alleviated early injury after ICH. Y. Wang et al. (2020) found that miRNA-183-5p levels were significantly reduced after ICH occurrence in mice. Injecting miRNA-183-5p agomir reduced oxidative stress and neuroinflammatory responses by inhibiting the expression of HO-1 mRNA in ICH mice (73).

#### 4.3.4 miRNAs in other inflammatory molecular pathways in ICH

Serum miR-21-5p levels were increased and were correlated with NIHSS scores. Inhibition of miR-21-5p could attenuate inflammatory damage, thereby alleviating neurological deficits after ICH via targeting with dual-specificity phosphatase 8 (DUSP8) (74).

It was reported that miR-132 enhanced cholinergic blockade of inflammatory responses and protected against ischemia-induced neuronal cell death by targeting acetylcholinesterase (AChE). Zhang et al. (2017) found that overexpression of miR-132 in the mice brain reduced neurological impairment and inflammatory damage. Consistently, the downregulation of miR-132 increased inflammation and apoptosis (76). In conclusion, the protective effect of miR-132 in ICH mouse models provides new opportunities for therapeutic interventions.

Wang et al. (2021) used collagenase-induced ICH mouse models and miR-144/451 knockout mice, and found that knockout of miR-144/451 increased TNF-α and IL-1β levels, and oxidative stress in ICH mice. Furthermore, the authors found that deletion of miR-144/451 suppressed the activity of the miR-451-14-3-3ζ-FoxO3 regulatory axis in ICH mice. Moreover, compared with miR-144, miR-451 was dominant in regulating ICH (98).

Overexpression of miR-590-5p significantly improved brain edema and neurological function and reduced neuroinflammation in ICH mice. Guo et al. (2018) showed that pelino-1 (Peli1) could be directly targeted by miR-590-5p. Overexpression of miR-590-5p significantly decreased Peli1 levels. Meanwhile, overexpression of Peli1 partially eliminated the inhibitory effect of miR-590-5p mimic (77).

In summary, multiple miRNAs have been demonstrated to hold therapeutic effects in ICH by targeting NF-κB signaling pathway, or directly binding to the 3’UTR of NLRP3 to inhibit its activation. Alternatively, miRNAs can activate Nrf2 signaling pathway to attenuate ICH-induced SBI.

### 4.4 miRNAs related neuroinflammation in TBI

Globally, nearly 300 in every 100,000 people suffer from TBI. According to the pathological process, TBI can be divided into two types: primary injury and secondary injury (99, 100). In addition to primary brain mechanical damage, a number of accompanying mechanisms lead to morbidity and mortality, including BBB dysfunction, mitochondrial dysfunction, inflammation, and excitotoxicity (101). Notably, brain inflammation in TBI patients has still been identified after a long time of being injury, leading to a persistent cognitive dysfunction (102). Neuronal survival and function are closely associated with inflammatory factors (103, 104). Therefore, targeting neuroinflammation may be important for improving TBI prognosis. It is reported that some miRNAs such as miR-21, miR-146, miR-155 and miR-223 could be induced by inflammatory stimuli. Moreover, multiple researches have shown that miR-21 played a role in neuroprotection and BBB integrity after TBI, indicating that miRNAs could affect the prognosis of TBI (105–107).

#### 4.4.1 miRNAs in NF-κB signaling pathway in TBI

Ge et al. (2016) found that miR-21-5p could regulate NF-κB signaling pathway to inhibit inflammation. *In vitro* experiments, miR-21-5p has been found to promote the activation of the angiopoietin 1 (Ang-1)/tyrosine kinase receptor 2 (Tie-2) pathway, thus promoting angiogenesis and repair of brain tissue. This study suggested that miR-21-5p not only inhibited inflammation by affecting the NF-κB signaling pathway, but was also closely related to apoptosis and vascular repair (75).

#### 4.4.2 miRNAs in AKT/mTOR signaling pathway in TBI

Li et al. (2020) showed that the upregulation of miR-23a inhibited neuroinflammation and improved long-term neural function. Overexpression of miR-23a inhibited caspase 3 activity and the release of inflammatory mediators, suggesting miR-23a could inhibit the apoptosis and inflammatory responses. Moreover, miR-23a could directly target the phosphatase and tensin homologue (PTEN). Overexpression of miR-23a activated the AKT/mTOR pathway in TBI mouse models, as evidenced by the increased levels of p-AKT and p-mTOR (78).

#### 4.4.3 miRNAs in MAPK signaling pathway in TBI

MiR-200b was reported to regulate the inflammatory response by modulating the MAPK pathway in microglia. The miRNA-200b levels were downregulated in activated microglia. Jadhav et al. (2014) found that the transcription factor c-Jun was the target of miR-200b, and inhibition of miR-200b in microglia enhanced JNK activity modulated microglial inflammatory process, and increasing neuronal apoptosis (79).

In summary, miR-200b targeted the cJun/MAPK signaling pathway and reduced the inflammatory response of activated microglia, indicating that miR-200b is a possible intervention target in chronic neuroinflammation. miRNA targeting neuroinflammation and its downstream effects, including NF-κB signaling pathway, MAPK signaling pathway and NLRP3 inflammasome, have important implications for improving the prognosis of TBI.

## 5 Therapy targeting miRNA in CNS diseases

**Figure 3:**
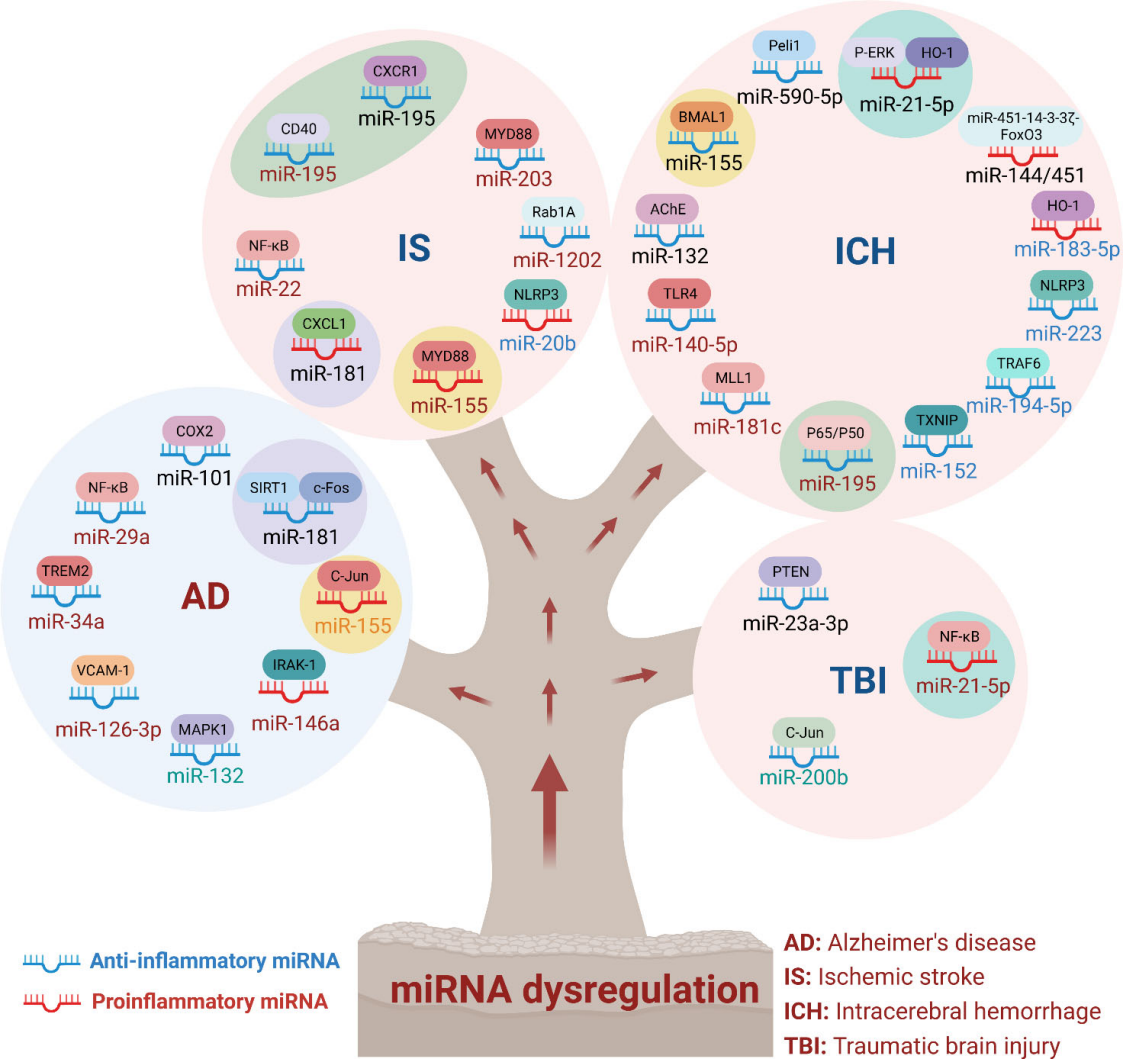
MiRNA dysregulation in neuronal system diseases. MiRNAs regulated a wide range of physiological and pathological processes. In immunoinflammatory responses in CNS diseases, miRNAs could be classified into anti-inflammatory and pro-inflammatory miRNAs. In this review, we summarized the targeted genes and signaling pathways of dysregulated miRNAs in different CNS diseases. As shown that miR-155 was involved in AD, IS and ICH. In addition, miR-21-5p, miR-181 and miR-195 were also involved in the inflammatory effects of multiple diseases. These results indicated that multiple miRNAs synergistically regulated neuroinflammation and affected the outcome of treatment of inflammation-mediated nervous system diseases.

Here, we summarized the role of miRNA in CNS diseases, and discussed the regulatory molecular mechanism of immune inflammatory response, involving the NF-κB signaling pathway, MAPK signaling pathway, and NLRP3 inflammasome. Interestingly, we found that some miRNAs played roles in more than one CNS disease, suggesting a critical role for these miRNAs in CNS diseases (**Figure 3**). For example, miR-21-5p, miR-155, miR181, and miR-195 were each involved in two or more CNS diseases. Mechanistically, MiR-155 regulated the c-Jun/NF-κB signaling pathway, is involved in AD, regulates the expression of TLR4 and MyD88 proteins, and is involved in IS. In hemorrhagic stroke, antagomir-155 inhibited miRNA-155 and promoted the expression of BMAL1, thus activated Nrf2 signaling pathway and alleviated brain injury after cerebral hemorrhage, suggested therapy targeting miRNA is benefit for the treatment of CNS diseases.

Furthermore, we summarized the therapies targeting miRNAs in CNS diseases (**Table 2**). Among them, antiretrovirals D4T reduced the activation of NLRP3 inflammasome, and inhibited the levels of downstream NLRP3 inflammasome molecules (53). Moreover, Klotho improve cellular inflammation by inhibiting cytokine release and upregulating miR-29a (36), suggesting that Klotho and the antiretroviral drug D4T may have beneficial therapeutic potential for attenuating neuroinflammation in AD. High-frequency repetitive transcranial magnetic stimulation (rTMS) inhibited microglia activation through the let-7b-5p/HMGA2/NF-κB signaling pathway, and protected against IS (108). In addition, some of the neuroprotective effects of DEX in several disease models were discovered to be mediated by various miRNAs, such as LPS-induced neuroinflammation, brain ischemic-reperfusion injury, and β-amyloid-induced dysfunction (111). Sevoflurane prevented ICH by miRNA-133b/FOXO4/BCL2 axis (109). Ginsenoside Rg1 ameliorated BBB breakdown and TBI by attenuating the release of macrophage-derived exosome miR-21 (110). In LPS-induced inflammation rat models, DEX was involved in the process of inflammation, autophagy, and apoptosis through the regulation of miR-21-5p and miR-155. In summary, these results suggest that a combination of drug therapy and specific miRNAs could play a broader and prospective role in the treatment of CNS diseases. At the same time, combining drug therapy with specific miRNAs to treat CNS diseases still has some limitations. For example, how to increase the stability of miRNA analogs *in vivo* application, and how to address the potential immunostimulatory effects. In addition, how to increase the concentration of combining drug therapy with specific miRNAs in lesion-specific area is another big challenge. Moreover, as usually more than one miRNA targeted for one gene in the CNS disease model, the combination and the therapeutic effects of drug-miRNA need to be further investigated.

**Table 2:** miRNA-related therapy in neurological diseases.

MicroRNA	Related disease	Target	Treat manner	Reference
miR-29a	AD	TNF-α/NF-кB	Klotho	(36)
miR-223-3p	AD	NLRP3	D4T	(53)
let-7b-5p	IS	HMGA2/NF-κB	rTMS	(108)
miR-195	IS	CD40/NF-κB, CX3CR1	Nanoparticle Intraventricular lentivirus injection	(64, 65)
miR-133b	ICH	FOXO4/BCL2 axis	Sevoflurane	(109)
miR-21	ICH	Rg1	exosome	(110)
miR-152	ICH	TXNIP/NLRP3	Intraventricular lentivirus injection	(70)
miR-194-5p	ICH	TRAF6/NLRP3	Injection of miR-194-5p agomir	(71)
miR-183-5p	ICH	HO-1/Nrf2	Injection of miR-183-5p agomir	(73)
Multiple miRNAs	ICH	–	DEX	(111)

## 6 Conclusion

Currently, the potential effects of miRNAs on neuroinflammatory regulation have been widely demonstrated. Moreover, inflammatory-miRNAs are significantly differentially expressed in the peripheral circulation in CNS patients. Therefore, miRNAs are promising biomarkers for the diagnosis and prognosis of these CNS diseases, and are potential therapeutic targets. Nevertheless, the role and regulatory mechanisms of inflammatory-miRNAs in neuroinflammation need to be further investigated. First, as most studies on miRNA in the treatment of chronic inflammation-mediated nervous system diseases focus on NF-κB signaling, MAPK signaling, and NLRP3 inflammasome pathways, more attention should be paid to discover new signaling pathways. Second, the ability of multiple miRNA combinations to treat diseases should be studied. For example, miR-29, miR-126-3p, and miR-146a may synergistically regulate neuroinflammation and affect treatment of inflammation-mediated nervous system diseases. Third, although some miRNAs are highly correlated with the inflammation-mediated nervous system diseases, whether these miRNA act as biomarkers in the diagnosis and progresses of these diseases are still to be validated.

## Author contributions

JZ reviewed the papers and wrote the paper. YT, AL and RG reviewed the manuscript and gave suggestions. JC supervised this research and wrote the paper. All authors contributed to the article and approved the submitted version.
